# Ultrathin polymeric films for interfacial passivation in wide band-gap perovskite solar cells

**DOI:** 10.1038/s41598-020-79348-1

**Published:** 2020-12-17

**Authors:** Parnian Ferdowsi, Efrain Ochoa-Martinez, Sandy Sanchez Alonso, Ullrich Steiner, Michael Saliba

**Affiliations:** 1 Adolphe Merkle Institute, University of Fribourg, 1700 Fribourg, Switzerland; 2 Laboratory of Photomolecular Science (LSPM), École Polytechnique Fédéral de Lausanne (EPFL), Station 6, 1015 Lausanne, Switzerland; 3 Institute for Photovoltaics (ipv), University of Stuttgart, Pfaffenwaldring 47, 70569 Stuttgart, Germany; 4 Helmholtz Young Investigator Group FRONTRUNNER, IEK5-Photovoltaik, Forschungszentrum Jülich, 52425 Jülich, Germany

**Keywords:** Solar cells, Applied optics

## Abstract

Wide band-gap perovskite solar cells have the potential for a relatively high output voltage and resilience in a degradation-inducing environment. Investigating the reasons why high voltages with adequate output power have not been realized yet is an underexplored part in perovskite research although it is of paramount interest for multijunction solar cells. One reason is interfacial carrier recombination that leads to reduced carrier lifetimes and voltage loss. To further improve the V_oc_ of methylammonium lead tri-bromide (MAPbBr_3_), that has a band-gap of 2.3 eV, interface passivation technique is an important strategy. Here we demonstrate two ultrathin passivation layers consisting of PCBM and PMMA, that can effectively passivate defects at the TiO_2_/perovskite and perovskite/spiro-OMeTAD interfaces, respectively. In addition, perovskite crystallization was investigated with the established anti-solvent method and the novel flash infrared annealing (FIRA) with and without passivation layers. These modifications significantly suppress interfacial recombination providing a pathway for improved V_OC_’s from 1.27 to 1.41 V using anti solvent and from 1.12 to 1.36 V using FIRA. Furthermore, we obtained more stable devices through passivation after 140 h where the device retained 70% of the initial performance value.

## Introduction

Organic–inorganic lead halide perovskite solar cells (PSCs) have attracted enormous interest in the past few years due to their ease of fabrication, low cost and performances rivaling the best thin-film photovoltaic devices^1–5^. The power conversion efficiency (PCE) of PSCs has leapt from 3.8%^6^ in 2009 to the current world record of 25.2%^7^ through improvements and engineering of solvents, interfaces and materials^8–11^. The perovskite material has an AB*X*_3_ formula that is comprised of an organic/inorganic monovalent cation, A (Cs^+^, methylammonium (MA^+^), or formamidinium (FA^+^)); a divalent metal, B (Pb^2+^); and a halide anion, *X* (Cl^–^, Br^–^, or I^–^)^5^.

To date, the classic prototype perovskite methylammonium lead triiodide (MAPbI_3_) has been one main focus in research due to its suitable band-gap for photovoltaics, near complete light absorption in thin films (< 1 μm) and its fast charge extraction rate. However, poor stability of MAPbI_3_, rapid degradation in humidity and non-world-record PCEs, despite numerous optimization efforts, hamper commercialization^1–3,12–15^.

Methylammonium lead tribromide (MAPbBr_3_) with a large band-gap of 2.3 eV, thus permitting a *V*_OC_ of nearly 2.0 V, can be a promising optoelectronic alternative to MAPbI_3_ (1.5 eV) due to its good charge transport and higher stability against air and moisture^3,16^.

MAPbBr_3_-PSCs received however less attention due to their high band-gaps that are unsuited for high-performance solar cells. For example, MAPbI_3_-PSCs have been reported with short circuit currents (*J*_SC_) of 20.5 mA/cm^2^ and open‐circuit voltages (*V*_OC_) of 1.26 V^17^, while, MAPbBr_3_ devices with *J*_SC_ = 4.8 mA/cm^2^ and *V*_OC_ = 1.37 V have been shown^18^. It is noteworthy that MAPbI_3_ devices have achieved high voltages compared to the narrow band-gap of the material. On the other hand, MAPbBr_3_ devices exhibited a *V*_OC_ of only 1.37 V compared to its wide band-gap of 2.3 eV. There is therefore still much potential to investigate the improvement of the open‐circuit voltage for wide band-gap PSCs. It is noteworthy that higher *V*_OC_s for MAPbBr_3_ (1.47 V^19^, 1.58^20^ and 1.61^21^) have been reported but these data is related to the structures which are different with our structure engineering. For instance, Noel et al. used the n–i–p device architecture with single-walled carbon nanotubes (SWCNTs) interpenetrated with spiro-OMeTAD as the hole extraction layer (FTO/SnO_2_/CH_3_NH_3_PbBr_3_/SWCNT/spiro-OMeTAD/Au) and they prepared the perovskite solution using acetonitrile/methylamine solvent system^19^. Wu et al. reported an inverted CH_3_NH_3_PbBr_3_ cell using Indene-C60 Bisadduct (ICBA) as an acceptor and they showed the importance of energy level matching of electron transport layer using ICBA^21^.

Furthermore, higher performance device based on MAPbBr_3_ (1.45 V, 9.75 mA/cm^2^, 8.7% PCE) has been reported by Sheng et al*.*, but they synthesized CH_3_NH_3_Br and then used a vapor-assisted method for depositing and fully crystalizing MAPbBr_3_ film on mesoporous TiO_2_^22^. In addition, Subhani et al. showed high performance for MAPbBr_3_ PSCs (1.40 V, 8.93 mA/ cm^2^, 9.54% PCE) utilizing diphenylether (DPE) as an anti-solvent^23^. Regarding device designing, there is a similar device design with our work that has been reported for different perovskite (Cs_0.07_Rb_0.03_FA_0.765_MA_0.135_PbI_2.55_Br_0.45_) by Peng et al.^24^. Our work is on MAPbBr_3_, with different energy level, and yet the same method works.

If realized, high open‐circuit voltages offer considerable opportunities for different applications such as water splitting, light-emitting devices, detectors and the pathway to an all-perovskite multijunction device that requires wide band-gap perovskites with high *V*_OC_’s^1,3,25^. Therefore, realizing a high band-gap perovskite material has been a neglected research area and future progress will depend on analyzing and closing the voltage gap in wide band-gap perovskites.

One reason for *V*_OC_ values that lie much below the band-gap of the material is recombination at the device interfaces, leading to reduced carrier lifetimes with consequent voltage loss. There are three main approaches to address this issue and to improve the *V*_OC_ of MAPbBr_3_ PSCs, such as reducing the defect density by adding dopants^5,26^, using alternative transport layers^27,28^, and interface passivation techniques that reduce the recombination at the perovskite/transport layer interfaces^4,29–31^.

One recent example for interface passivation are polymeric layers that reduce the surface roughness, recombination at the interface and improve stability by blocking metal electrode migration into the perovskite layer at elevated temperatures. This strategy is used for both the interface of electron-transporting layer (ETL)/perovskite and the hole-transporting layer (HTL)/perovskite. For example, it has been shown that the addition of a solution-processed PMMA (Poly(methyl methacrylate)) layer at perovskite/HTL interface improves the photovoltaic performance and stability^29^. Also, it was demonstrated that an ultrathin passivation layer consisting of a PMMA:PCBM ([6,6]-phenyl-C_61_-butyric acid methyl ester) mixture can benefit the perovskite/TiO_2_ interface through passivating the defects and suppressing interfacial recombination^4^.

Here, we demonstrate ultrathin passivation layers consisting of PMMA, PCBM and a PMMA/PCBM mixture that can effectively passivate defects at the perovskite/ ETL and perovskite/HTL interfaces, respectively. These modifications significantly suppress interfacial recombination, providing a pathway for improved *V*_OC_’s.

In addition, crystallization and film-formation are important key components in high quality perovskite materials. In this context, we have experimentally investigated the perovskite crystallization with the established anti-solvent and the “novel flash infrared annealing (FIRA)”^32,33^ methods, with and without passivation layers. This is the first demonstration of FIRA for MAPbBr_3_, revealing an altered film morphology and therefore a novel strategy to control the crystallization process. Additionally, interesting approaches have been investigated by using high-intensity near-infrared radiation technique to rapidly anneal perovskite layers^34–36^. Troughton et al*.*, proposed a new method of post annealing perovskite films within a short period of time, i.e. under 2.5 s using near-infrared radiation^34^. In another work^35^, Baker et al*.,* reduced the heating time of the mesoporous layers from two hours to 30 s. Lately, Martin et al*.*, reported fabrication of flexible perovskite solar cells through rapid thermal annealing technique to reduce post-deposition time from 150 to 14 s^36^.

Given the advantages of using the FIRA method for the manufacture of photovoltaic devices, such as environmentally and rapid processing^37,38^, combining the use of polymeric passivation and the FIRA method is a step forward to improve the efficiency and stability of the wide-band-gap PSCs. However, to the best of our knowledge, the combination of the passivation technique of the mp-TiO_2_/perovskite interface and the use of a rapid infrared annealing process to crystallize the precursor has not been investigated so far.

## Results

### Device characterization

**Figure 1:**
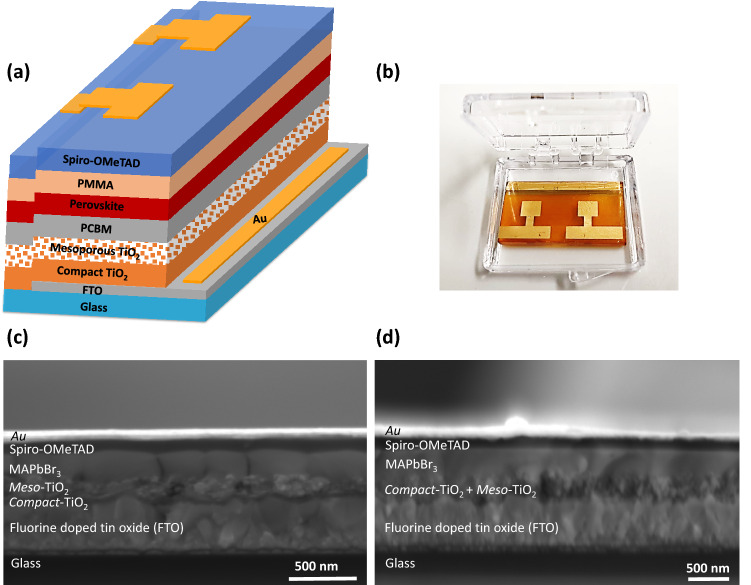
(**a**) Schematic of the device structure, (**b**) Fabricated device (MAPbBr_3_), (**c**) SEM cross-section of a perovskite solar cell; the MAPbBr_3_ layer was deposited by the anti-solvent technique, and (**d**) a FIRA-annealed MAPbBr_3_ layer with the structure FTO/c-TiO_2_/meso-TiO_2_/MAPbBr_3_/Spiro/Au.

Methylammonium lead bromide perovskite (MAPbBr_3_) was fabricated on mesoporous TiO_2_ layers by the anti-solvent and FIRA method. Figure 1a,b show the device architecture of a PSC and fabricated device with thin PCBM and PMMA layers on top of the mesoporous TiO_2_ and the perovskite layer, respectively.

Cross-sectional SEM images of anti-solvent and FIRA devices are shown in Fig. 1c,d. Top view SEM images of a PSC, using FIRA, are presented in the Supporting Information (SI) in Fig. S1.

Figure 1c reveals that the typical thicknesses of the compact TiO_2_, mesoporous TiO_2_, MAPbBr_3_, spiro-OMeTAD and gold layers were approximately 67, 100, 336, 137 and 80 nm, respectively. For the FIRA device in Fig. 1d, the compact and mesoporous TiO_2_ layer (combined), perovskite and spiro thickness were about 185, 330 and 120 nm, respectively, and those of the PCBM and PMMA were 12 nm $\pm 3$ and 1.5 nm $\pm 1$ according to AFM measurements (Fig. S2). The results indicate that the PMMA layer is sufficiently thin to permit the tunneling of charge carriers to the electrodes. They are sufficiently thick to fill conformally cover the interfaces and they may passivate the defects the perovskite top surface.

To evaluate the passivation performance of the ultrathin polymeric layer at the interface of perovskite and HTL, we prepared cells with and without thin PMMA (0.1 mg/ml) films at the MAPbBr_3_/HTL interface through the anti-solvent method and compare the device performances of these cells. The *V*_OC_ and PCE of the solar cells improved from 1.31 $\pm$ 0.07 to 1.34 $\pm$ 0.04 V on average by the addition of a thin PMMA layer (Supporting Information, Fig. S3).

Furthermore, to evaluate the effect of a second passivation layer at the ETL/perovskite interface, we prepared cells with different PMMA:PCBM ratios (w/w): a control cell (no passivation layer), 1:3, 1:5 and 1:10. The obtained data showed no clear trend (Fig. S4). We then prepared cells with pure PMMA (1 mg/ml), pure PCBM (5 mg/ml) and 1:20 (PMMA:PCBM) films at the ETL/perovskite interface in addition of PMMA (0.1 mg/ml) on top of the perovskite. All other cell fabrication steps were kept the same. The results showed that pure PCBM at the bottom of MAPbBr_3_ in addition of pure PMMA on top, improved the *V*_OC_ significantly from 1.25 $\pm$ 0.04 to 1.39 $\pm$ 0.01 V, on average, as well as the PCE from 4.9 $\pm$ 0.33 to 6.1 $\pm$ 0.40% compared to the devices with one passivation layer (Fig. S5).

**Figure 2:**
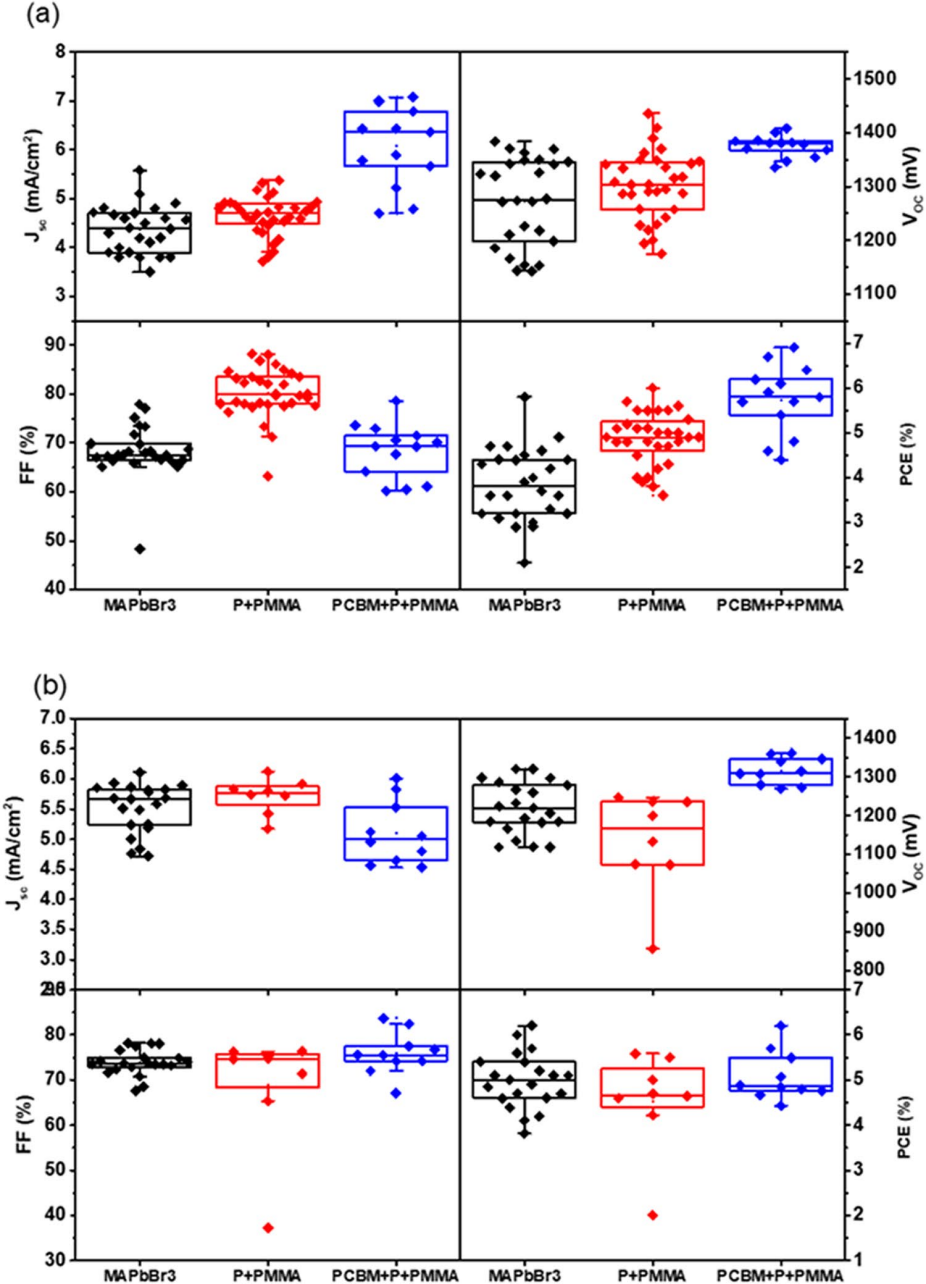
Statistical distribution of the photovoltaic parameters for cells with/without PMMA and PCBM passivation layers: (**a**) anti-solvent deposited MAPbBr_3_ layers, (**b**) FIRA-annealed MAPbBr_3_ layers.

Figure 2 shows the device statistics (short circuit current, open circuit voltage, fill factor (FF) and PCE) of the control devices (no passivation) and passivated devices (P + PMMA, PCBM + P + PMMA, where P denotes the perovskite layer) collected for different batches using the anti-solvent (a) and FIRA (b) methods. We note improvements in all device parameters through passivation (Fig. 2a) and observe that two ultrathin layers at the top and bottom of perovskite have the most positive effect on the performance (5.74 $\pm$ 0.59%) compared to the devices with only one passivation layer (4.67 $\pm$ 0.45%) and no passivation layer (3.85 $\pm 0.66$%). In addition, the voltage improvement for FIRA devices through passivation is shown in Fig. 2b. It is important to mention that FIRA devices required a separate optimization in the IR annealing time at 1.4 s and a solvent ratio of 3:1 for DMF:DMSO (Figs. S6 and S7).

**Figure 3:**
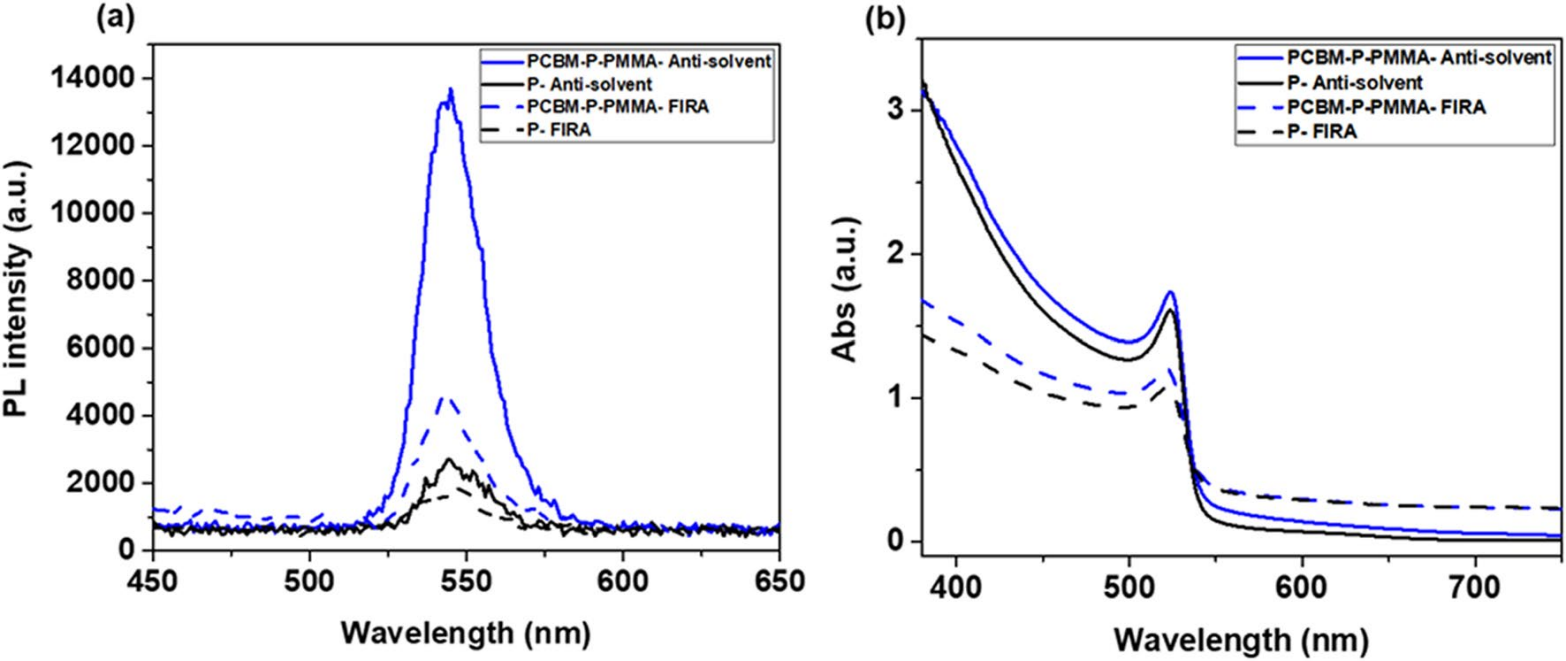
(**a**) Photoluminescence (PL) and (**b**) absorption spectra of cells with the structure FTO/c-TiO_2_/meso-TiO_2_/PCBM/MAPbBr_3_/PMMA and FTO/c-TiO_2_/meso-TiO_2_/MAPbBr_3_.

To further characterize the passivation performance of the intermediate layers, we also investigated the photo-luminescence (PL) intensity of the different cells with the structure FTO/c-TiO_2_/mesoporous-TiO_2_/PCBM/MAPbBr_3_/PMMA and FTO/c-TiO_2_/mesoporous-TiO_2_/MAPbBr_3_. We present the UV–vis and PL data comparing devices with anti-solvent processed (solid line) and FIRA annealed (dashed line) MAPbBr_3_ layers in Fig. 3.

As previously reported^4^, the devices with the highest *V*_OC_ have the highest PL intensity. As shown in Fig. 2, devices with two passivation layers result in a considerable *V*_OC_ improvement. This agrees with the PL data for devices that were prepared with both the anti-solvent and FIRA method. The PL intensity of passivated devices is higher than that of the non-passivated ones (Fig. 3a).

Therefore, we conclude that the increase in PL intensity for our best passivated cells suggests that the surface recombination at the ETL/perovskite and perovskite/HTL interfaces is significantly suppressed by the addition of the thin intermediate layers, resulting in an increased *V*_OC_. The PL spectra shows peaks positioned at 545 and 542 nm for passivated devices by anti-solvent and FIRA, respectively, and at 544 and 546 for non- passivated ones, respectively.

The absorption spectra of cells is shown in Fig. 3b. Devices prepared through the anti-solvent method exhibited local absorption maxima of 524 nm and FIRA-annealed devices had maximum absorption peaks positioned at 522 nm. As shown in Fig. 3b, passivated cells have higher absorption compared to non-passivated ones which agrees with the PL data.

**Figure 4:**
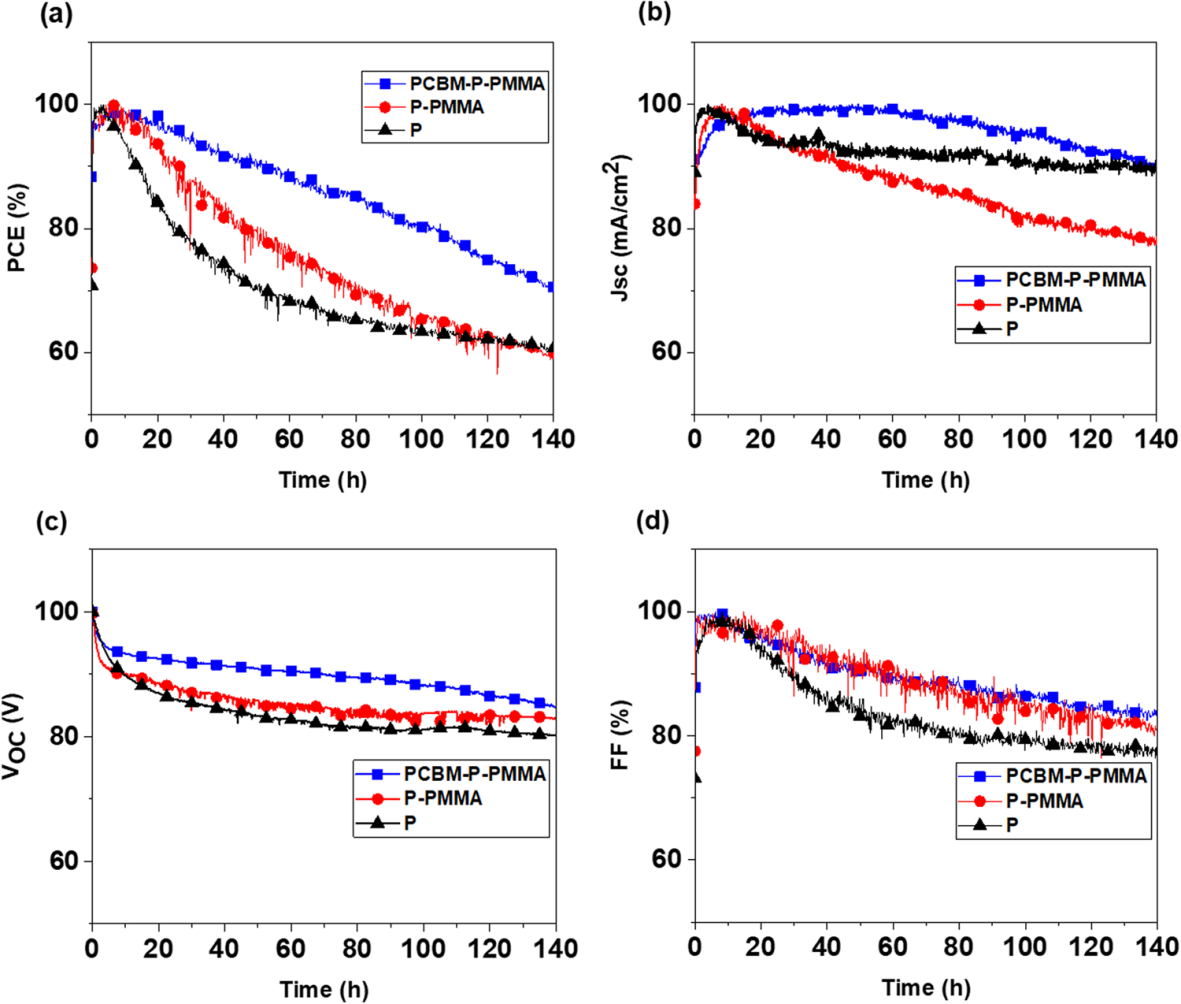
Variation of main normalized photovoltaic parameters (**a**) output power, (**b**) short-circuit photocurrent, (**c**) open circuit voltage, and (d) fill factor of anti-solvent-manufactured devices with two passivation layers (blue curve), one passivation layers (red curve) and without passivation layers (black curve) aged at room temperature after 140 h in a nitrogen atmosphere.

**Figure 5:**
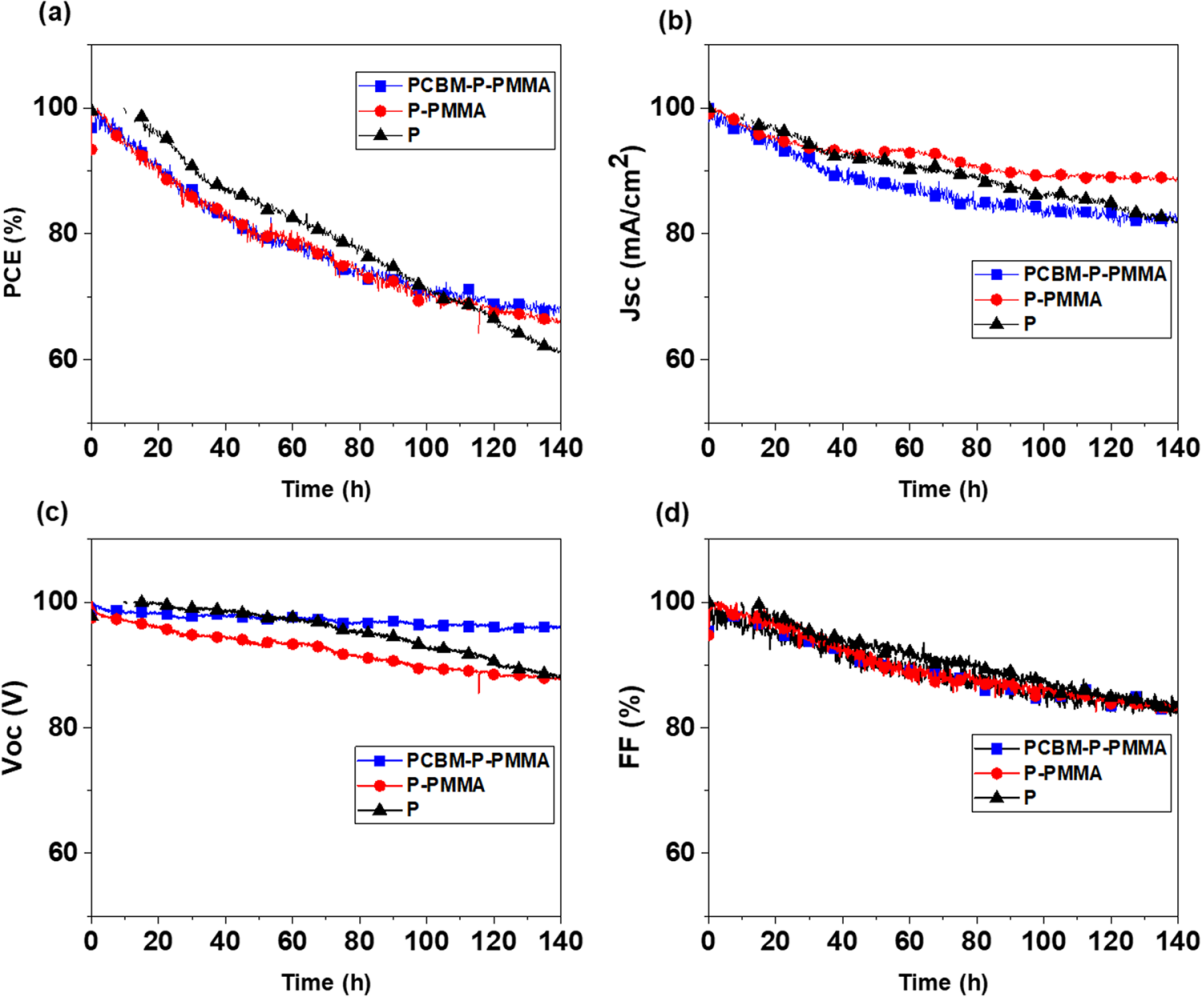
Variation of main normalized photovoltaic parameters (**a**) output power, (**b**) short-circuit photocurrent, (**c**) open circuit voltage, and (**d**) fill factor of FIRA-annealed MAPbBr_3_ devices with two passivation layers (blue curve), one passivation layers (red curve) and without passivation layers (black curve) aged at room temperature after 140 h in a nitrogen atmosphere.

Furthermore, in Figs. 4 and 5 we investigated long-term device stability of the devices in a nitrogen atmosphere held at room temperature under constant illumination of one sun. A *JV* scan was taken periodically to extract the observed device parameters. Figures 4 and 5 shows the variation of the normalized output power, short-circuit photocurrent, open circuit voltage and fill factor of three devices using no passivation, one and two passivation layers, comparing anti-solvent and FIRA-annealed devices, respectively.

Figure 4a shows that without passivation layers, the loss in cell performance is significant during the initial hours. In contrast, the two passivated devices showed only small losses and retained 70% of the initial performance after 140 h of continuous illumination. The PCE of the non-passivated device dropped quickly after 20 h to 83% of its initial performance, then decreased with a gentle slope from 80% at 25 h to around 67% at 70 h, and then stayed relatively constant reaching 60% of the initial performance after 140 h. The device with only one PMMA passivation layer also decreased to 60% of its initial performance after 140 h.

The variation of *J*_SC_ exhibited a similar trend and all the devices were followed by a slow recovery during ca. 10 h of light soaking and the device with two passivation layers reached to 92% of its initial current (Fig. 4b).

For all the devices, the *V*_OC_ remained nearly constant (Fig. 4c). It is noted that the obtained results show a further improvement in the *V*_OC_ using two passivation layers compare to the reported highest value with the same structure in literature (1.37 V)^18^.

Fill factor variations reveal similar trends for the output power for no, one and two passivation layers reaching 78, 82 and 84% of their initial FF, respectively (Fig. 4d).

The stability data highlights the significance of the device architecture and the important role of passivation layers at the perovskite interfaces for the long-term stability.

Li et al. proved the passivation effect of the surface trap states on device stability^39^. These two-passivation layers decrease the trap density of states thus prevent unwanted trap-assisted electron–hole recombination at the interfaces and create physical barriers between transportation layers and the perovskite film. In addition to that, the PCBM passivation was deposited at the interface of TiO_2_ and perovskite film to physically separate ETL from perovskite layer. This leads to an improvement in the device stability by enhancing oxygen tolerance and avoiding the rapid degradation which can be caused by the UV sensitive TiO_2_ surface^39,40^. Moreover, further improvement in device stability is achieved due to the fact that PMMA can behave as a shielding layer for the perovskite film against humidity and oxygen under ambient conditions^29^.

In Fig. 5a, for the FIRA processed devices the PCE of the non-passivated device dropped more quickly than the other two devices and reached around 60% of the initial performance after 140 h. The devices with one and two passivation layers showed similar trends and decreased with a gentle slope to a more than 70% PSC decline at 95 h, and then stayed relatively constant reaching around 70% of the initial performance after 140 h. Notably, PSCs with passivation thin films remain more stable against the device with a non-passivated film.

The *J*_SC_ of the unpassivated device and the one with two passivation layers reached 82% of the initial current after 140 h, while the one with one passivation layer showed a bit more stable trend and reached to 89% of the initial *J*_SC_ after 140 h (Fig. 5b).

The variation of *V*_OC_ of all devices exhibited more or less similar trends until 90 h and then, devices with no and one passivation layer decreased with a gentle slope to nearly 90% of the initial voltage after 140 h. The device with two passivation layers remained nearly constant and showed 97% of its initial *V*_OC_ after 140 h (Fig. 5c).

Fill factor variations reveal similar trends for all devices and reached close to 90% at 70 h, and then stayed relatively constant reaching 83% of their initial FF after 140 h (Fig. 5d).

**Figure 6:**
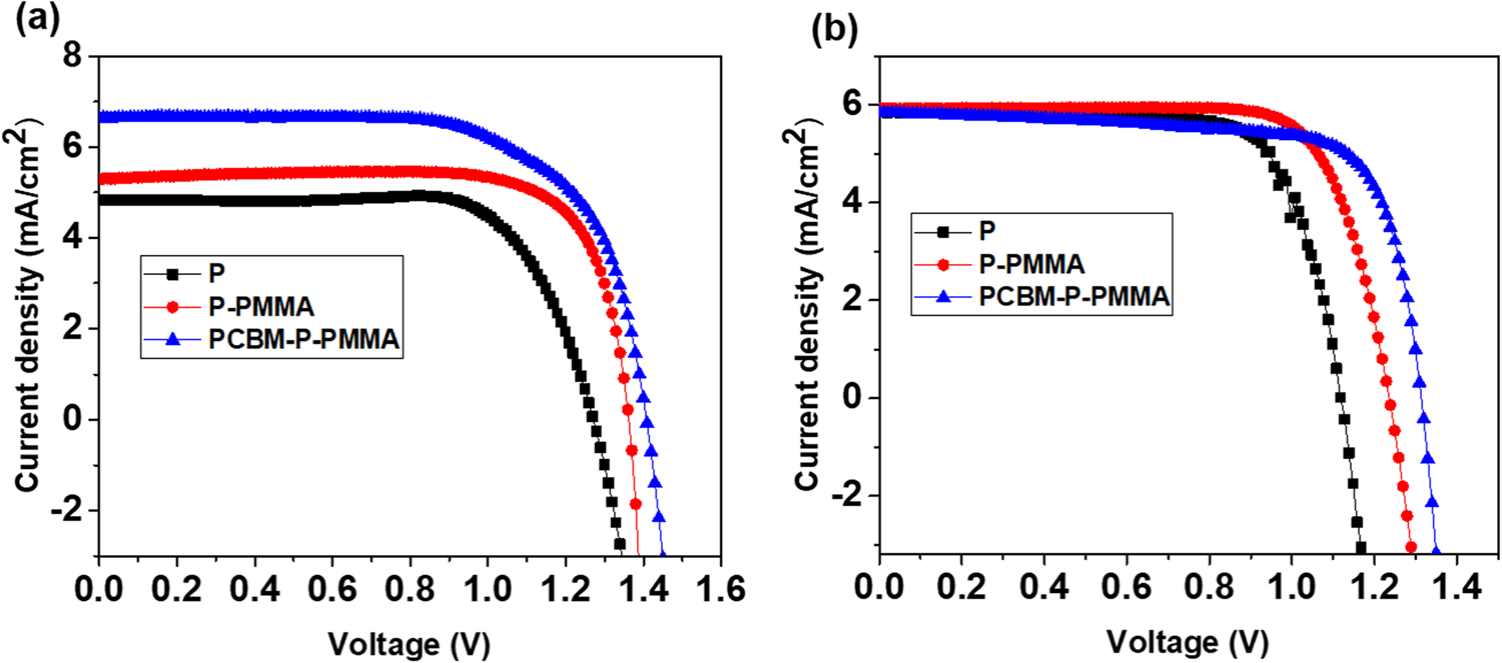
Current density–voltage curves of devices with and without PMMA and PCBM passivation layers, made by (**a**) the anti- solvent method and (**b**) FIRA-annealing. The scan rate was 20 mV s^−1^.

Typical photocurrent density–voltage (*J–V*) curves of a PSC containing no passivation layer, one passivation layer (0.1 mg/mL PMMA coated on MAPbBr_3_) and two passivation layers (5 mg/mL PCBM at ETL/MAPbBr_3_ interface in addition of top polymeric layer) made by the anti-solvent method and by FIRA-annealing are shown in Fig. 6. The corresponding photovoltaic parameters are collected in Table 1.

**Table 1:** Photovoltaic parameters of the devices with/without passivation layers through anti-solvent and FIRA.

Samples	V_OC_ (V)		J_SC_ (mA/cm^2^)		FF		PCE (%)	
	Anti-solvent	FIRA	Anti-solvent	FIRA	Anti-solvent	FIRA	Anti-solvent	FIRA
P	1.27	1.12	4.82	5.85	73.5	74.1	4.50	4.85
P-PMMA	1.36	1.24	5.29	5.92	78.2	76.3	5.64	5.58
PCBM-P-PMMA	1.41	1.31	6.63	5.83	67.7	74.2	6.32	5.68

Figure 6a shows the performance of devices that were prepared by the anti-solvent method. The control cell had a PCE of 4.5%, *V*_OC_ = 1.27 V, *J*_SC_ = 4.82 mA cm^−2^ and FF = 73.5. In contrast, the cell with the two passivation layers achieved a PCE of 6.32%, *V*_OC_ = 1.41 V, *J*_SC_ = 6.63 mA cm^−2^ and FF = 67.7%. From the obtained data, it is clear that the *V*_OC_ has improved due to the presence of one polymeric layer and that two passivation layers have a much more positive effect.

FIRA devices showed a similar trend and a significantly improved *V*_OC_ can be seen for this kind of device (Fig. 6b).

Therefore, ultrathin passivation layers can effectively passivate the ETL/MAPbBr3/HTL interfaces, leading to a considerable reduction in charge recombination and a dramatic increase in *V*_OC_ from 1.27 and 1.12 V (non-passivated) to 1.41 V and 1.31 V (two passivated) for devices manufactured by the anti-solvent method and FIRA-annealing, respectively. The passivating effects at the interfaces is an essential factor to improve the performance of perovskite solar cells. In the presence of intermediate layers, i.e. PCBM and PMMA, at the bottom and top surface of the perovskite layer, trapping effects of the photo generated electron–hole pairs are reduced. These states are typically generated by crystal defects and pinholes of the deposited perovskite layer induced by substrate roughness and grain boundaries. The PCBM and PMMA layers reduce the charge recombination rate and the forbidden energy states transitions at the interfaces by passivating interface and near-interface traps.

Furthermore, Photovoltaic parameters of champion control and passivated old devices were remeasured from both reverse and forward scans under 20 mV s^−1^ scan rate (Supporting Information (SI) in Figs. S8-S9 and Table S2).

## Discussion

In this work, we show that ultrathin polymeric passivation layers consisting of PCBM and PMMA can effectively passivate the ETL/MAPbBr_3_/HTL interfaces of wide band-gap perovskite solar cells, leading to a significant increasing in *V*_*OC*_ from 1.27 V (non-passivated) to 1.36 V (one-passivation layer) and 1.41 V (two passivation layers) using anti solvent method. The results showed that there is a potential to further improve the *V*_OC_ and increase the electron lifetime through passivation, which was successful to reduce the interfacial charge recombination. Furthermore, this work, for the first time used FIRA technique for MAPbBr_3_ to control the perovskite crystallization process. We investigated the effect of passivation layers for FIRA-annealed devices and the obtained results show an *V*_OC_ improvement from 1.12 V (non-passivated) to 1.24 V (one-passivation layer) and 1.31 V (two passivation layers). Given the advantages of FIRA, i.e. environmentally friendly and rapid processing, this is a highly promising method for the scale-up of PSC manufacture. The long-term stability investigation show that the variation of *V*_OC_ for devices with two passivation layers remained nearly constant and reached to around 86% and 97% of their initial *V*_OC_ in devices made by the anti-solvent method and FIRA-annealing, respectively.

## Methods

### Substrate preparation and Li-doping TiO_2_

Fluorine-doped tin oxide (FTO) pre-etched substrates (Sigma-Aldrich, TEC-7) were cleaned by sonication in 2% Hellmanex water solution, isopropyl alcohol (IPA) and ethanol, respectively. Then all the substrates were further cleaned with plasma treatment for 10 min. A TiO_2_ compact layer was deposited on FTO by spray pyrolysis, using oxygen as a carrier gas, at 450 °C from a precursor solution of titanium diisopropoxide bis(acetylacetonate) stock solution (75 wt.% in isopropanol) in anhydrous ethanol. Then, a mesoporous TiO_2_ layer was deposited by spin coating using a 30 nm particle paste (Dyesol 30 NR-D) diluted in ethanol. After the spin coating, the substrates were sintered with a ramped temperature profile, keeping the temperature at 125, 225, 375, 450, and 25 °C for 5, 5, 5, and 30 min, respectively, with 5, 15, 5, and 5 min ramp duration between each temperature^1,30,33^.

Li-treatment of mesoporous TiO_2_ is done by spin coating a 10 mg mL^−1^ solution of Li-TFSI in acetonitrile. After cooling down, the substrates were transferred in a nitrogen atmosphere glove box to deposit the perovskite films^41^.

### PCBM and PMMA precursor solution

For the ETL side, the PCBM precursor solution was prepared by dissolving 5 mg/mL PCBM (Sigma Aldrich, 99.5%) in chlorobenzene.

The PCBM:PMMA ($x$:1) precursor solution was prepared by dissolving $x$ mg PCBM and 1 mg PMMA into 1 mL chlorobenzene.

The solution was spin coated statically on the substrates at 4000 rpm for 30 s.

For the HTL side, the PMMA precursor solution was prepared by dissolving 0.1 mg PMMA in 1 mL chlorobenzene and solution was spin coated dynamically on a rotating substrate at 4000 rpm for 30 s.

### Perovskite precursor solution and film preparation

The organic cation salts were purchased from Greatcellsolar; the lead compounds from TCI; DMF and DMSO from Sigma-Aldrich. The following formulations were composed by mixing the appropriate amounts.

#### Anti-solvent

The MAPbBr_3_ solution was prepared from a precursor solution containing MABr and PbBr_2_ (1.5 M) in anhydrous DMF:DMSO 4:1 (v:v). (The PbBr_2_ solution was added to MABr powder; PbBr_2_: MABr (1:1); Final perovskite solution (1.22 M)).

The perovskite solution was spin coated at 3000 rpm for 30 s. Around 10 s prior to the end of the program, 100 μL of chlorobenzene was injected on the spinning substrate. Films turned yellow immediately after spin coating. The substrates were then annealed (usually at 100 °C) for 45 min in a nitrogen filled glove box.

#### FIRA

The MAPbBr_3_ solution was prepared from a precursor solution containing MABr and PbBr_2_ (1.4 M) in anhydrous DMF:DMSO 3:1 (v:v). (The PbBr_2_ solution was added to MABr powder; PbBr_2_: MABr (1:1); Final perovskite solution (1.30 M)).

The films were prepared by spin-coating of the perovskite solution in a single step at 4000 rpm for 10 s. in the next step, the prepared substrates were then IR irradiated for 1.4 s in the FIRA oven and were kept there for 20 additional seconds before removal. The hole process was done in a standard fume hood^33^.

### Hole transporting layer and top electrode

After the perovskite annealing, the substrates were cooled down and a Spiro-OMeTAD (Lumtec) solution (70 mM in chlorobenzene) was spin coated at 4000 rpm for 20 s. Spiro-OMeTAD was doped with bis(trifluoromethylsulfonyl)imide lithium salt (Li-TFSI, SigmaAldrich) and 4-tert-Butylpyridine (TBP, SigmaAldrich) in the molar ratio of 0.5 and 3.3 for Li-TFSI and TBP, respectively.

Finally, gold with 80 nm thickness was thermally evaporated under high vacuum as top electrode^33^.

### Characterization

All devices were tested under simulated solar irradiation (100 mW/ cm^2^, AM 1.5G) using a solar simulator from ABET Technologies (Model Sun 2000) with a xenon arc lamp, and the solar cell response was recorded using a Metrohm PGSTAT302N Autolab. The light intensity was calibrated using a silicon reference cell from ReRa Solutions (KG5 filtered)^33^. Current–voltage curves were measured in a scan rate of 20 mV s^−1^ at reverse and forward bias. The cells were masked with a black metal mask (0.09936 cm^2^) to fix the active area and reduce the influence of the scattered light. UV–Vis measurements were performed on Shimadzu UV-2401PC Spectrophotometer. Scanning electron microscopy (SEM) images were obtained with a Tescan Mira3 LM FE. Photoluminescence measurements on perovskite films were performed using a Horiba Fluorolog FL 3–22 spectrometer. Atomic force microscopy (AFM) was performed on JPK NanoWizard II. Optical microscopy was done on ZEISS Axio Scope.A1.

## Supplementary Information

Supplementary Information.
