# Supplementary material for: Ultrathin polymeric films for interfacial passivation in wide band-gap perovskite solar cells
Source: Sci Rep. 2020 Dec 17;10:22260. doi: 10.1038/s41598-020-79348-1 (PMC7746738; doi:10.1038/s41598-020-79348-1)
Supplement: Supplementary file 1 — Supplementary Information. [file 41598_2020_79348_MOESM1_ESM.docx]

**Ultrathin Polymeric Films for Interfacial Passivation in Wide Band-gap Perovskite Solar Cells**

Parnian Ferdowsi ^1^, Efrain Ochoa-Martinez ^1^, Sandy Sanchez Alonso ^2^, Ullrich Steiner^1^ and Michael Saliba^3,^*

1 Adolphe Merkle Institute, University of Fribourg, CH 1700, Fribourg, Switzerland.

^2^ Laboratory of Photomolecular Science (LSPM), École Polytechnique Fédéral de Lausanne (EPFL), Station 6, 1015 Lausanne, Switzerland.

^3^Institute for Photovoltaics (ipv), University of Stuttgart, Pfaffenwaldring 47, 70569 Stuttgart, Germany. (Email: [michael.saliba@ipv.uni-stuttgart.de](mailto:michael.saliba@ipv.uni-stuttgart.de), [miliba@gmail.com](mailto:miliba@gmail.com))

**Table of content**

| **Supplementary Figure S1.** Top view SEM image of MAPbBr_3_ through FIRA | Page 2 |
| --- | --- |
| **Supplementary Figure S2.** 2-D, 3-D AFM image and the corresponding line profiling analysis of (a) PCBM, and (b) PMMA-coated on Si substrate. Note that the PMMA and PCBM layer for profile-AFM measurement was deposited by spin-coating 0.1 mg/mL PMMA and 5 mg/mL PCBM in CB at 4000 rpm for 30 s on the top of Si substrate | Page 2 |
| **Supplementary Figure S3.** Statistical distribution of the photovoltaic parameters for cells with/without PMMA (0.1 mg/ml) at the MAPbBr_3_/HTL interface through anti-solvent | Page 3 |
| **Supplementary Figure S4.** Statistical distribution of the photovoltaic parameters for cells with/without PMMA, PMMA: PCBM passivation layers through anti-solvent: “P” is the control device and the other three devices include two passivation layers, on top (PMMA, 0.1 mg/ml) and bottom of perovskite (PMMA (1 mg/ml): PCBM (5 mg/ml), different ratio)) | Page 3 |
| **Supplementary Figure S5.** Statistical distribution of the photovoltaic parameters for cells with pure PMMA (1 mg/ml), pure PCBM (5 mg/ml) and PMMA: PCBM (1:20) at the bottom of perovskite in addition of PMMA (0.1 mg/ml) on top of the perovskite through anti-solvent: (PMMA on top is the control device and the other three devices include two passivation layers, on top and bottom of perovskite) | Page 4 |
| **Supplementary Figure S6.** IR annealing time optimization for FIRA | Page 4 |
| **Supplementary Figure S7.** Different solvent ratio (DMF: DMSO) to optimize the perovskite solution for FIRA | Page 5 |
| **Supplementary Figure S8.** Current density-voltage curves of old devices with and without PMMA and PCBM passivation layers, made by anti- solvent method, from both reverse and forward scans under 20 mV s^−1^ scan rate. | Page 5 |
| **Supplementary Figure S9.** Current density-voltage curves of old devices with and without PMMA and PCBM passivation layers, made by FIRA-annealing, from both reverse and forward scans under 20 mV s^−1^ scan rate. | Page 6 |
|  |  |
| **Supplementary Table S1.** PCBM and PMMA thickness at several points of layer on the top of Si substrate from AFM data | Page 6 |
|  |  |
| **Supplementary Table S2.** Photovoltaic parameters of the old devices with/without passivation layers through anti-solvent and FIRA, from both reverse and forward scans under 20 mV s^−1^ scan rate | Page 7 |


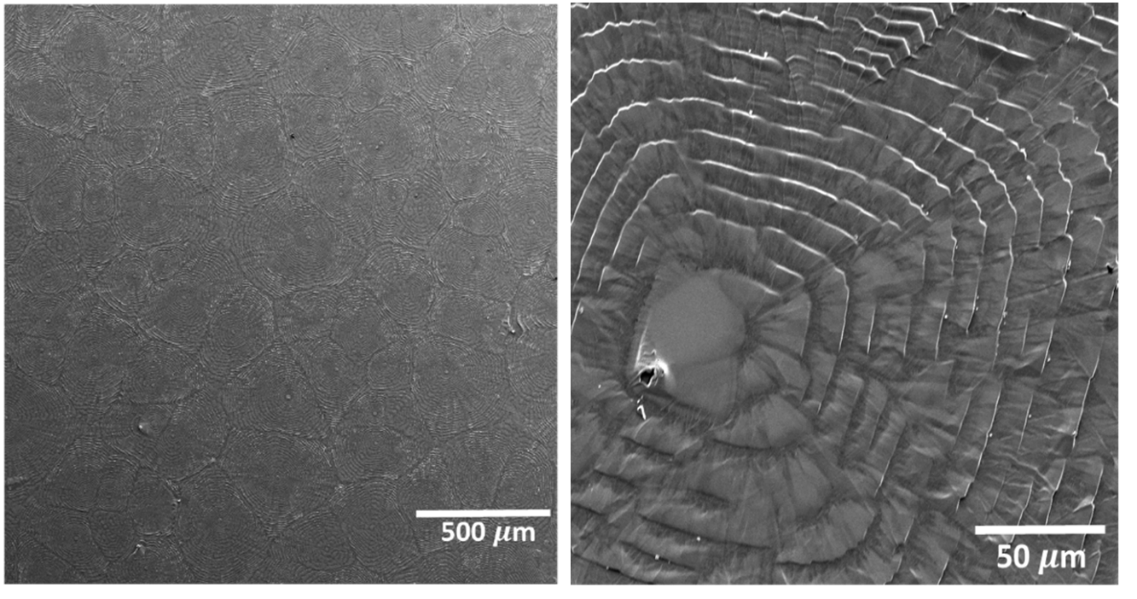


**Supplementary Figure S1.** Top view SEM image of MAPbBr_3_ through FIRA


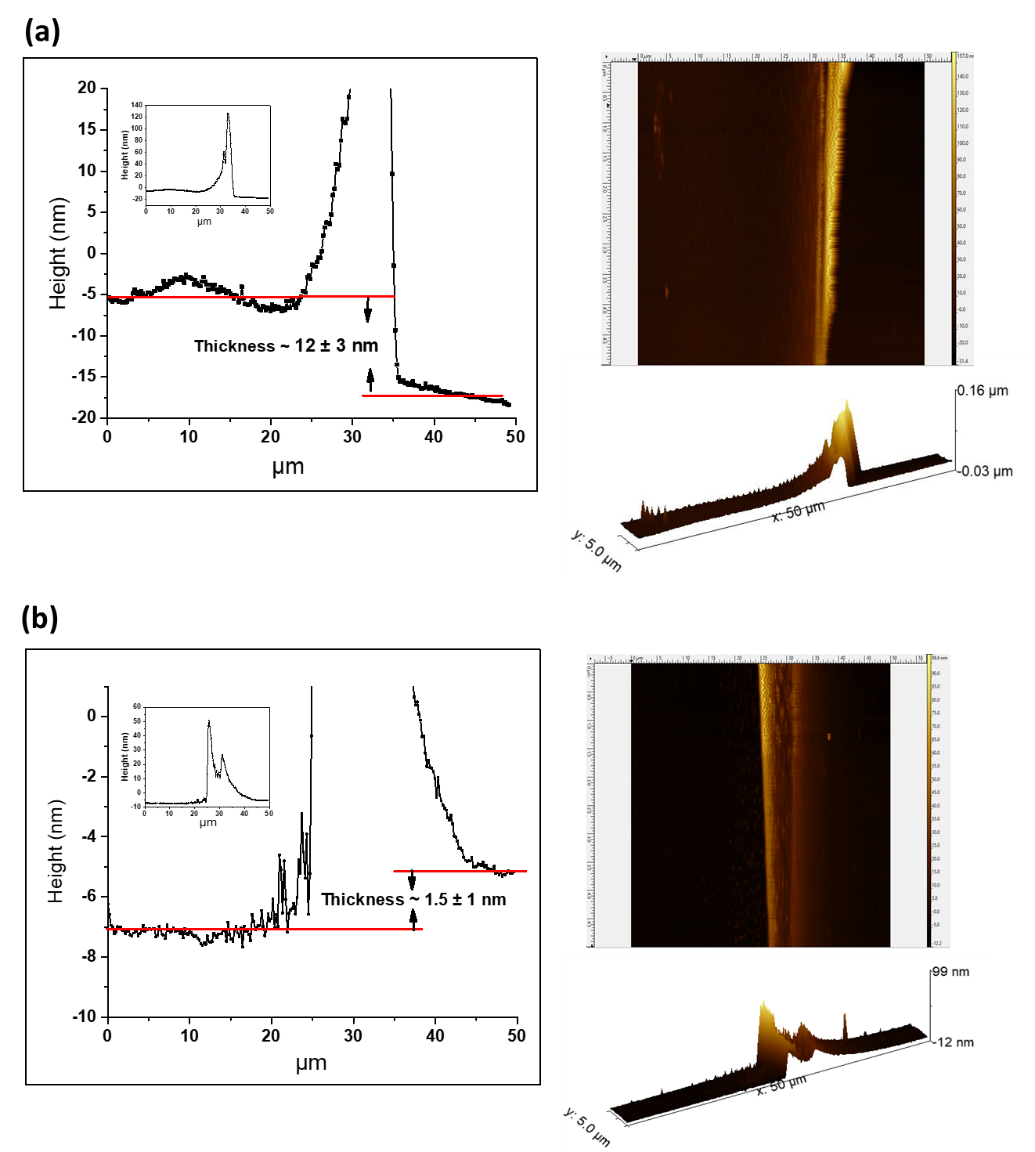


**Supplementary Figure S2.** 2-D, 3-D AFM image and the corresponding line profiling analysis of (a) PCBM, and (b) PMMA-coated on Si substrate with 50-picometer resolution. Note that the PMMA and PCBM layer for profile-AFM measurement was deposited by spin-coating 0.1 mg/mL PMMA and 5 mg/mL PCBM in CB at 4000 rpm for 30 s on the top of Si substrate.

**Supplementary Figure S3.** Statistical distribution of the photovoltaic parameters for cells with/without PMMA (0.1 mg/ml) at the MAPbBr_3_/HTL interface through anti-solvent.

**Supplementary Figure S4.** Statistical distribution of the photovoltaic parameters for cells with/without PMMA, PMMA: PCBM passivation layers through anti-solvent: “P” is the control device and the other three devices include two passivation layers, on top (PMMA, 0.1 mg/ml) and bottom of perovskite (PMMA (1 mg/ml): PCBM (5 mg/ml), different ratio)).

**Supplementary Figure S5.** Statistical distribution of the photovoltaic parameters for cells with pure PMMA (1 mg/ml), pure PCBM (5 mg/ml) and PMMA: PCBM (1:20) at the bottom of perovskite in addition of PMMA (0.1 mg/ml) on top of the perovskite through anti-solvent: (PMMA on top is the control device and the other three devices include two passivation layers, on top and bottom of perovskite).

**Supplementary Figure S6.** IR annealing time optimization for FIRA


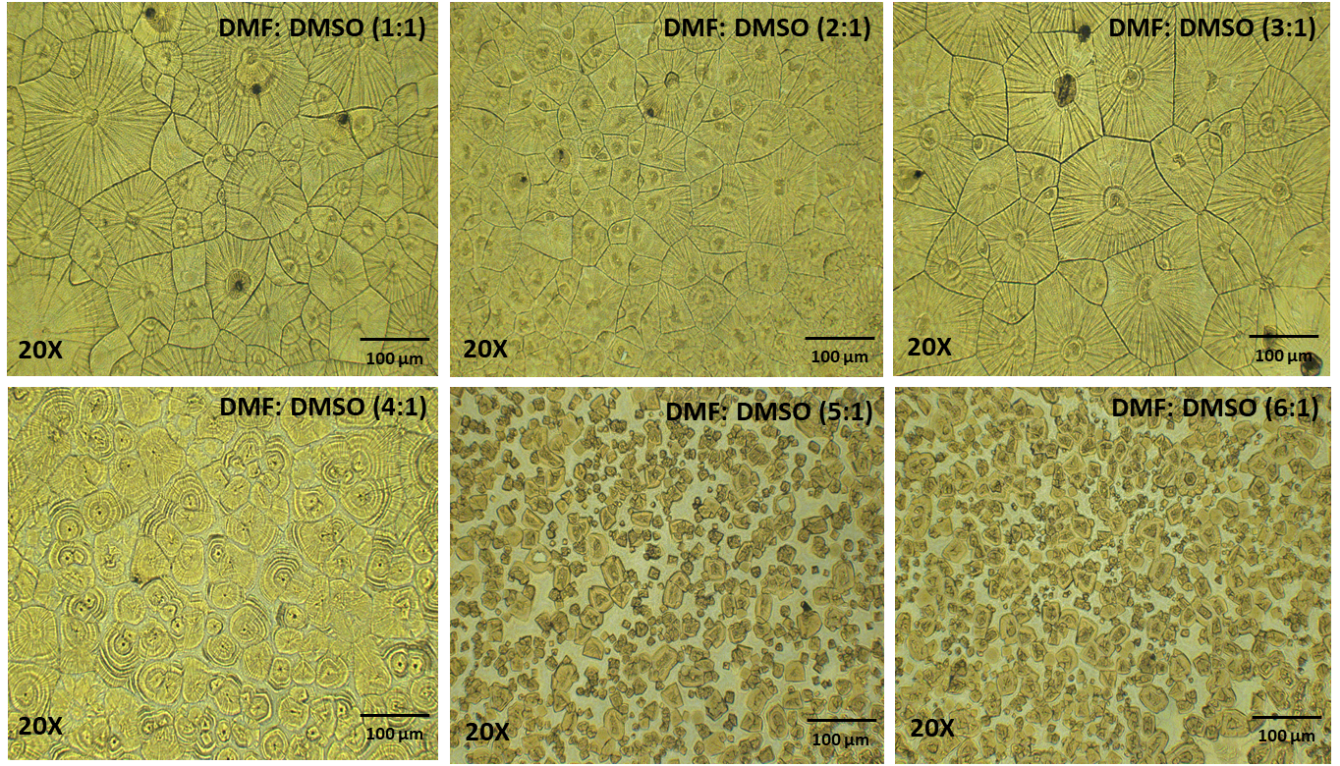


**Supplementary Figure S7.** Different solvent ratio (DMF: DMSO) to optimize the perovskite solution for FIRA

The devices were remeasured at 20 mV s^−1^ from both the reverse and forward scans. The results showed that the devices with passivation layers shows negligible hysteresis between reverse and forward J–V scans, achieving a 71.4 and 89.8% PCE of their initial performance for anti-solvent and FIRA devices, respectively.


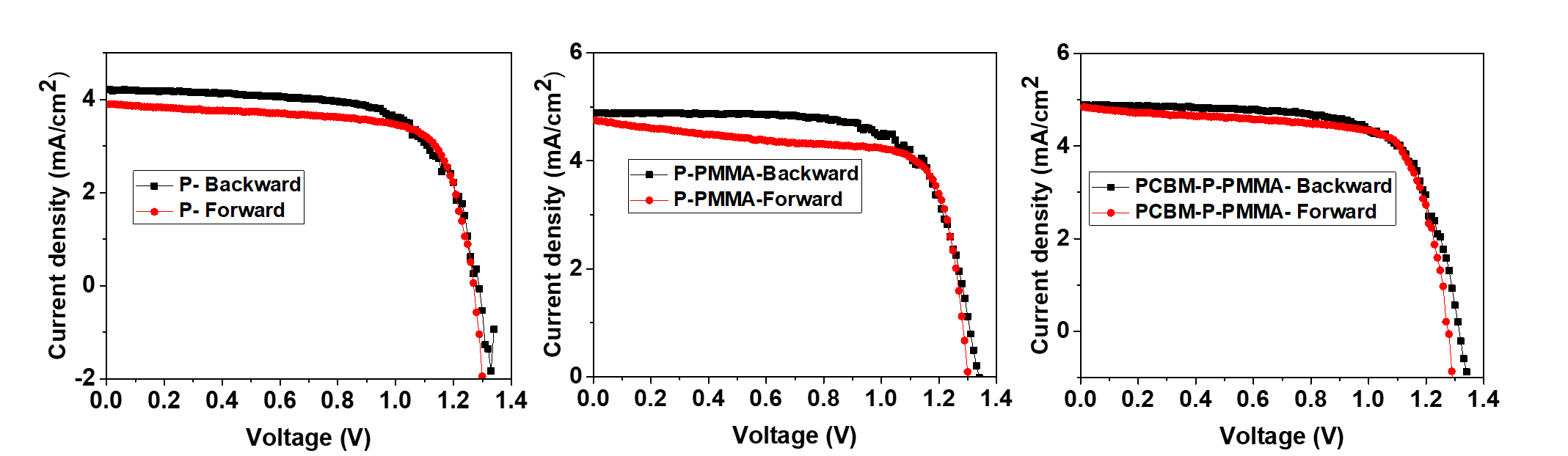


**Supplementary Figure S8.** Current density-voltage curves of old devices with and without PMMA and PCBM passivation layers, made by anti- solvent method, from both reverse and forward scans under 20 mV s^−1^ scan rate.


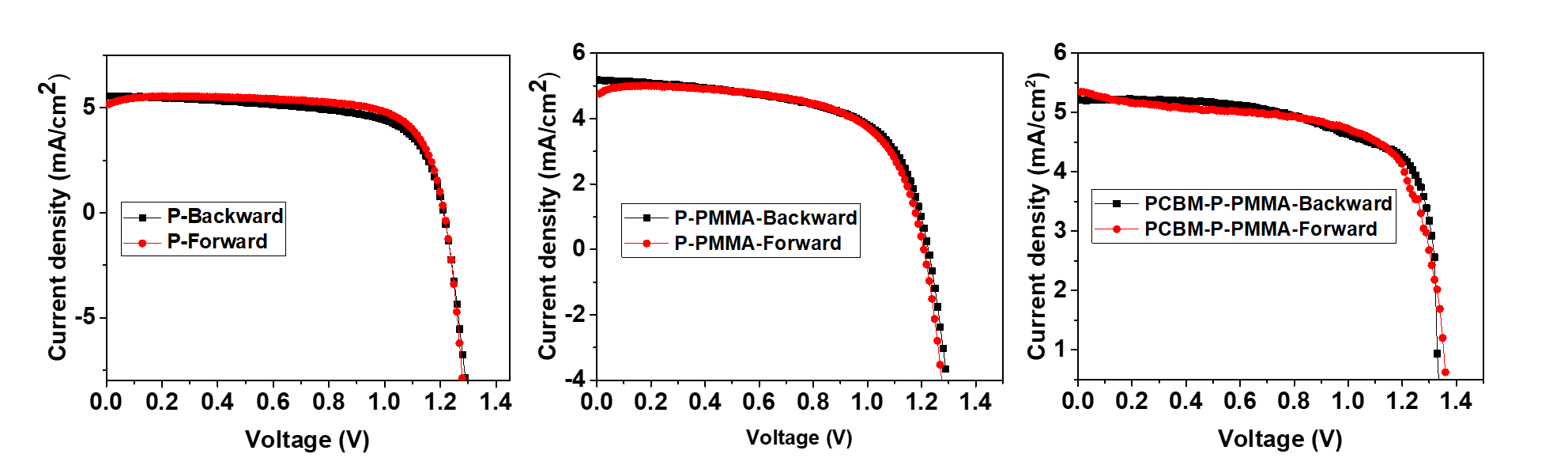


**Supplementary Figure S9.** Current density-voltage curves of old devices with and without PMMA and PCBM passivation layers, made by FIRA-annealing, from both reverse and forward scans under 20 mV s^−1^ scan rate.

| **Supplementary Table S1.** PCBM and PMMA thickness at several points of layer on the top of Si substrate from AFM data | | | | | |
| --- | --- | --- | --- | --- | --- |
| PCBM | Left | Backward | Inside | 15nm | AVERAGE= 12 $\pm3$ |
|  |  |  | Outside | 10nm |  |
|  |  | Forward | Inside | 8nm |  |
|  |  |  | Outside | 15nm |  |
|  | Right | Backward | Inside | 10nm |  |
|  |  |  | Outside | 15nm |  |
|  |  | Forward | Inside | 8nm |  |
|  |  |  | Outside | 15nm |  |
| PMMA | Left | Backward | Inside | >1nm | AVERAGE= 1.5$\pm1$ |
|  |  |  | Outside | 1nm |  |
|  |  | Forward | Inside | 2.5nm |  |
|  |  |  | Outside | 1nm |  |
|  | Right | Backward | Inside | 1nm |  |
|  |  |  | Outside | 2.5nm |  |
|  |  | Forward | Inside | 1 |  |
|  |  |  | Outside | 2.5nm |  |

| **Supplementary Table S2.** Photovoltaic parameters of the old devices with/without passivation layers through anti-solvent and FIRA, from both reverse and forward scans under 20 mV s^−1^ scan rate | | | | | | | | | | |
| --- | --- | --- | --- | --- | --- | --- | --- | --- | --- | --- |
|  | **Sample**  **(Anti-solvent)** | **V_OC_ (V)** | | **J_SC_ (mA/cm^2^)** | | **FF** | | **PCE (%)** | | PCE degradation percentage (%) |
|  |  | RS | FS | RS | FS | RS | FS | RS | FS | RS |
| Anti-solvent | P | 1.29 | 1.27 | 4.21 | 3.90 | 67.7 | 72.1 | 3.67 | 3.57 | 81.6 |
|  | P-PMMA | 1.33 | 1.29 | 4.68 | 4.57 | 70.4 | 72.4 | 4.37 | 4.26 | 77.5 |
|  | PCBM-P-PMMA | 1.32 | 1.28 | 4.89 | 4.84 | 70.2 | 72.7 | 4.51 | 4.50 | 71.4 |
| FIRA | P | 1.23 | 1.21 | 5.18 | 4.76 | 60.5 | 66.3 | 3.84 | 3.81 | 79.2 |
|  | P-PMMA | 1.21 | 1.21 | 5.56 | 5.18 | 65.6 | 76.6 | 4.42 | 4.81 | 79.2 |
|  | PCBM-P-PMMA | 1.34 | 1.37 | 5.22 | 5.25 | 72.9 | 68.6 | 5.10 | 5.03 | 89.8 |
